# Phenolic Profile, Antioxidant and Enzyme Inhibitory Activities of Leaves from Two *Cassia* and Two *Senna* Species

**DOI:** 10.3390/molecules27175590

**Published:** 2022-08-30

**Authors:** Haifa A. A. Omer, Giovanni Caprioli, Doaa Abouelenein, Ahmed M. Mustafa, Abdullahi Ibrahim Uba, Gunes Ak, Refiye Beyza Ozturk, Gokhan Zengin, Sakina Yagi

**Affiliations:** 1 Department of Botany, Faculty of Science, Sudan University of Science and Technology (SUST), Khartoum P.O. Box 407, Sudan; 2 Department of Botany, Faculty of Science, University of Khartoum, Khartoum P.O. Box 321, Sudan; 3 Chemistry Interdisciplinary Project (CHIP), School of Pharmacy, University of Camerino, Via Madonna delle Carceri, 62032 Camerino, Italy; 4 Department of Molecular Biology and Genetics, Faculty of Engineering and Natural Sciences, Kadir Has University, Istanbul 34083, Turkey; 5 Physiology and Biochemistry Research Laboratory, Department of Biology, Science Faculty, Selcuk University, Konya 42130, Turkey

**Keywords:** *Cassia* spp., *Senna* spp., phenolic compounds, antioxidant, enzyme inhibition

## Abstract

Several species within the genera *Cassia* or *Senna* have a treasure of traditional medicines worldwide and can be a promising source of bioactive molecules. The objective of the present study was to evaluate the phenolic content and antioxidant and enzyme inhibition activities of leaf methanolic extracts of *C. fistula* L., *C. grandis* L., *S. alexandrina* Mill., and *S. italica* Mill. The two *Cassia* spp. contained higher total polyphenolic content (42.23–49.75 mg GAE/g) than the two *Senna* spp., and *C. fistula* had significantly (*p* ˂ 0.05) the highest concentration. On the other hand, the *Senna* spp. showed higher total flavonoid content (41.47–59.24 mg rutin equivalent per g of extract) than that found in the two *Cassia* spp., and *S. alexandrina* significantly (*p* ˂ 0.05) accumulated the highest amount. HPLC–MS/MS analysis of 38 selected bioactive compounds showed that the majority of compounds were identified in the four species, but with sharp variations in their concentrations. *C. fistula* was dominated by epicatechin (8928.75 µg/g), *C. grandis* by kaempferol-3-glucoside (47,360.04 µg/g), while rutin was the major compound in *S. italica* (17,285.02 µg/g) and *S. alexandrina* (6381.85). The methanolic extracts of the two *Cassia* species exerted significantly (*p* ˂ 0.05) higher antiradical activity, metal reducing capacity, and total antioxidant activity than that recorded from the two *Senna* species’ methanolic extracts, and *C. fistula* displayed significantly (*p* ˂ 0.05) the highest values. *C. grandis* significantly (*p* ˂ 0.05) exhibited the highest metal chelating power. The results of the enzyme inhibition activity showed that the four species possessed anti-AChE activity, and the highest value, but not significantly (*p* ≥ 0.05) different from those obtained by the two *Cassia* spp., was exerted by *S*. *alexandrina*. The *Cassia* spp. exhibited significantly (*p* ˂ 0.05) higher anti-BChE and anti-Tyr properties than the *Senna* spp., and *C. grandise* revealed significantly (*p* ˂ 0.05) the highest values. *C. grandise* revealed significantly (*p* ˂ 0.05) the highest α- amylase inhibition, while the four species had more or less the same effect against the α-glucosidase enzyme. Multivariate analysis and in silico studies showed that many of the identified phenols may play key roles as antioxidant and enzyme inhibitory properties. Thus, these *Cassia* and *Senna* species could be a promising source of natural bioactive agents with beneficial effects for human health.

## 1. Introduction

Bioactive compounds derived from natural resources, especially from plants, provide huge potentials against a broad spectrum of pharmacological targets with beneficial effects on the human health care system [1]. Metabolomics has evolved in recent years as a comprehensive approach to examine a complex mixture of molecules that may be associated with observations obtained through biological activity tests without the need for isolation procedures; consequently, aiding the development of natural product-derived therapeutic agents [2]. A number of phytoconstituents with scientific evidences of their pharmacological potency have been discovered over the last few years. From the total approved drugs (1562 drugs) by the USFDA between 1981 and 2014, 4% were pure natural products, 9.1% were herbal mixtures, 21% were derived from natural products, and 4% were synthetic drugs prepared by exploring the pharmacophores of natural products [3]. Medicinal plants, traditionally used in the management of ailments, are gaining increasing attention from scientists worldwide with the view to identify new drug candidates to address diseases [4].

The utilization of herbal products with low side effects is always beneficial for the improvement of health [5]. *Cassia* is a large genus of around 500 species of flowering plants in the family Leguminaceae/Fabaceae and is widely distributed throughout Africa, Asia, and North and South America [6]. Taxonomically, and due to many morphological complexes, the genus *Cassia* was segregated into three allied genera, namely *Cassia* L. Sensu stricto, *Chamaecrista* Moench, and *Senna* miller. The two genera *Senna* and *Cassia* have economic importance for ornamental, nutritive, and medicinal purposes, and also wood production and degraded area restoration [7]. Many species have been used extensively for their laxative effects caused by sennosides present in these two genera [8]. Previous studies on the chemical composition of both genera have identified flavonoids, anthraquinones, stilbenoids, pentacyclic triterpenes, pyrones, and alkaloids [9,10]. Pharmacologically, they possess hepatoprotective, antipyretic, anti-inflammatory, leukotriene inhibition, antioxidant, wound healing, anticancer, antidiabetic, and antimicrobial activities among others [7,11].

There are 23 species belonging to the genus *Cassia* in Sudan, 16 of which are indigenous and 7 are exotic. They are distributed between the genera *Senna* (12 species), *Cassia* (8 species) and *chamaecrista* (3 species) [12]. The majority of species are used in traditional medicine for the treatment of different ailments. For example, *Senna alexandrina* Mill. (Syn. *Cassia senna* L. and *C. acutifolia* Del) is used to cure constipation, flatulence, diabetes, stomach ache, skin allergies, and sores [13]. *Senna italica* Mill. (Syn. (*Cassia obovata* Collad. and *C. italica* (Mill.) Lam. ex Andrews)) is used to treat dysentery, acne, rheumatic pain, and as a laxative [14]. *Cassia fistula* L. is used against jaundice, piles, rheumatism, ulcers, ring worms, and also externally to cure skin eruptions, eczema, and other skin diseases [15]. *Cassia grandis* L. is used against worms and intestinal parasites, and to treat stomach and respiratory problems, and infected wounds [16]. Generally, few studies on the enzyme inhibition property and antioxidant activity using different assays of the above four mentioned species have been reported. Considering all of these issues and in an attempt to explore plant-based alternative solutions for humans’ welfare, the objective of this study is to determine the composition of the phenolic content of *C. fistula, C. grandis, S. alexandrina*, and *S. italica* as well as their antioxidant activity and enzyme inhibitory properties against cholinesterase, amylase, glucosidase, and tyrosinase.

## 2. Results and Discussion

### 2.1. Total Polyphenolic and Flavonoid Contents

Phenolic compounds are well known for their important health benefits; they possess diverse pharmacological activities, such as antioxidant, antimicrobial, and anticancer among others [17]. In the present study, the total polyphenolic and flavonoid contents of the leaf methanolic extracts of the investigated *Cassia* and *Senna* spp. were determined and the results are presented in Table 1. The two *Cassia* spp. contained higher total polyphenolic content (42.23–49.75 mg GAE/g) than the two *Senna* spp. (21.54–22.25 mg GAE/g), and *C. fistula* had significantly (*p* ˂ 0.05) the highest concentration. On the other hand, the *Senna* spp. showed higher total flavonoid content (41.47–59.24 mg RE/g) than that found in the two *Cassia* spp., and *S. alexandrina* significantly (*p* ˂ 0.05) accumulated the highest amount. Additionally, the two *Senna* spp. showed higher amounts of total flavonoid content that their respective total polyphenolic content. Variation in polyphenolic and flavonoid contents of the studied species from values reported by Tzekiat and Chiang [18] for *C. fistula*, Fuentes et al. [19] for *C. grandis*, Laghari et al. [20] for *S. alexandrina*, and by Gololo et al. [21] for *S. italica* could be attributed to different factors among them; genetics factors, age and organ of plant, geographical areas and climatic conditions for the growth of the plant, as well as extraction solvent and methods [7]. Nevertheless, the results of the present study were in agreement with these previous studies in that *Cassia* and *Senna* spp. accumulated high total contents of polyphenolic and flavonoids.

### 2.2. Chemical Phenolic Profile by HPLC–MS/MS

Methanolic extracts of the studied *Cassia* and *Senna* spp. were analyzed by HPLC–MS/MS to examine their chemical profiles. The target compounds were quantified using the HPLC–MS/MS technique after the acquisition settings of the dynamic MRM mode were optimized. Table S1 lists the chosen ion transitions and mass spectrometer parameters for each molecule. Thirty-eight reference compounds belonging to different phenolic classes were used and the results are depicted in Table 2. The majority of compounds were identified in the four species. *C. grandis* (65,214.79 µg/g) followed by *C. fistula* (38,897.47 µg/g) accumulated higher total individual contents than *S. italica* (25,019.64 µg/g) and *S. alexandrina* (21,798.71 µg/g). The highest value of *C. grandis* was attributed to the remarkably high content in Kaempferol-3-glucoside (47,360.04 µg/g). However, it was also observed that there were sharp variations in the concentrations of compounds in the four species. For example, the major compounds: epicatechin (8928.75 µg/g), catechin (361.85 µg/g), procyanidin b2 (1767.78 µg/g), and quercitrin (1563.25 µg/g) in *C. fistula* were present in very high concentrations compared to other species. The same observation was noted in *C. grandis* for Kaempferol-3-glucoside (47,360.04 µg/g), phloridzin (2106.51 µg/g), ellagic acid (116.46 µg/g), and phloretin (16.65 µg/g), while rutin (17,285.02 µg/g) and quercetin (1207.88 µg/g) were in high concentration in *S. italica*. Malvidin-3-galactoside (14.19 µg/g) and petunidin-3-glucoside (2.51 µg/g) were only detected in *S. alexandrina*. Other compounds, such as isoquercitrin (1042.35–6526.84 µg/g) and hyperoside (1293.52–8270.74 µg/g), were relatively distributed in considerable amounts in the four species. Overall, *C. fistula* was dominated by epicatechin, hyperoside, and isoquercitrin, while *C. grandis* was dominated by kaempferol-3-glucoside, trans-cinnamic acid and phloridzin. Rutin and p-coumaric acid were the major compounds in *S. alexandrina*. However, *S. italica* was characterized by a remarkable highest concentration of rutin. Previous phytochemical studies were mainly performed on *C. fistula* and the two *Senna* species and have mainly reported the isolation of anthraquinones, anthranoides, anthrones, and flavonoids [7]. Few studies report the chemical constituents of *C. grandis* including the isolation of aloe-emodin from the leaf [22], 1,3,4-trihydroxy-6,7,8-trimethoxy-2-methyl anthraquinone-3-O-β-D- Glucopyranoside from the pod [23], and emodin-9-anthrone from the stem [24]. However, it is worth mentioning that this is the first detailed study on the phenolic profiles of these four species and this untargeted metabolomics approach would enable us to identify the composition of extracts to integrate biological knowledge, and, consequently, pinpoint pharmaceutical candidates [2].

### 2.3. Antioxidant Activity

Antioxidant agents can reduce the damaging effects of free radicals and may, therefore, play a protective role to attenuate oxidative stress-related diseases [25]. In the present study, six complementary in vitro DPPH, ABTS, CUPRAC, FRAP, MCA, and phosphomolybdenum assays were adopted to examine the antioxidant activity of the four species, and the results are presented in Table 3. The methanolic extracts of the two *Cassia* species exerted significantly (*p* ˂ 0.05) higher anti-DPPH and -ABTS radical activity than that recorded from the two *Senna* species’ methanolic extracts. Moreover, the antiradical activity of *C. fistula* from the DPPH and ABTS assays was, respectively, 2.2- and 2.8-fold higher than that obtained from *C. grandis*. Furthermore, the metal reducing capacity of the methanolic extracts of the two *Cassia* species was significantly (*p* ˂ 0.05) higher than that obtained from two *Senna* species’ methanolic extracts and the four species had higher Cu^++^ reducing capacity than the Fe^+++^ one. Again, the Fe^+++^ and Cu^++^ reducing activity of *C. fistula* was, respectively, 1.4- and 1.7-fold higher than that exerted by *C. grandis*. The *Senna* species were not significantly (*p* ≥ 0.05) different in their metal reducing capacity. The two *Cassia* species and *S. italica* revealed metal chelating power (11.38–8.11 mg EDTAE/g), and the highest and least values were recorded from *C. grandis* and *C. fistula*, respectively. The results of the total antioxidant activity from the phosphomolybdenum assay showed that the *Cassia* spp. exhibited significantly (*p* ˂ 0.05) higher activity than the *Senna* spp., and *C. fistula* recorded the highest value (1.94 mmol TE/g). On comparing these results with previous reports in the literature, it was observed that previous studies on these species were mainly evaluating their antiradical property. For example, Tzekiat and Chiang [18] reported that leaf samples of *C. fistula* under different age classes showed high anti-DPPH activity (IC_50_ 0.040–0.050 g/mL). Laghari et al. [20] showed that the *S. alexandrina* leaf exerted anti-DPPH activity (IC_50_ 3.6–7.4 mg/L) using different extraction techniques with the best results obtained from the microwave technique. A recent study on *S. italica* aerial parts showed that the isolated quercetin (95.8%) and rutin (94.2%) possessed potent antiradical activity from the DPPH assay [26]. Another recent study on *C. grandis* reported on the antioxidant activity of the fruit, showing values of 6.48 µg/g 0.34 mg/g from the DPPH and FRAP assays, respectively [19].

However, the significant antioxidant activity of the four species in the present study could be attributed to their total phenolic content and composition. *S. fistula* had the highest total polyphenolics content and it was observed that when considering the total content of all bioactive compounds without kaempferol-3-glucoside, *A. fistula* also recorded the highest total individual content (35,499.35 µg/g), which might significantly reflect on its highest antioxidant activity in most assays (5/6). This was in agreement with previous reports that demonstrated a good correlation between total bioactive compounds and antioxidant activity [17,27,28]. Moreover, many of the identified compounds in the present study were well known for their high antioxidant activity, such as epicatechin [29], caffeic acid [30], glycosides of kaempferol [31], delphindin 3,5 diglucoside [32], catechin [33], cinnamic acid [34], and hyperoside [35] among others.

### 2.4. Enzyme Inhibition Activity

Enzyme inhibitors play a great physiological and medical significance in the treatment of many diseases, including diabetes mellitus, hyperpigmentation, and central nervous system-related diseases [36]. In this context, the enzyme inhibitory property of the methanolic extracts of the investigated *Cassia* and *Senna* spp. was evaluated against AChE, BChE, Tyr, α-glucosidase, and α-amylase enzymes. The results are depicted in Table 4. The four species had anti-AChE activity in the range of 1.71–2.41mg GALAE/g, and the highest value, but not significantly (*p* ≥ 0.05) different from those obtained by two *Cassia* spp, was exerted by *S*. *alexandrina*. In addition, the *Cassia* spp. exhibited significantly (*p* ˂ 0.05) higher anti-BChE (0.95–1.15 mg GALAE/g) and anti-Tyr (34.51–46.58 mg KAE/g) properties than the *Senna* spp., and *C. grandise* revealed significantly (*p* ˂ 0.05) the highest values. Regarding their inhibition capacity against the two enzymes associated with diabetes, all four investigated species inhibited the α-glucosidase enzyme (1.43−1.45 mmol ACAE/g) better than the α- amylase (0.37–0.48 mmol ACAE/g) one. *C. grandise* revealed significantly (*p* ˂ 0.05) the highest α- amylase inhibition, while the four species had more or less the same effect against the α-glucosidase enzyme. It is worth mentioning that this is the first study on the enzyme inhibitory activity of the four species against these enzymes except for *C. grandis*, where a previous study on the pulp of the fruit reported its α-glucosidase inhibition [37]. Alhawarri et al. [38] reported that the high anti-AChE activity of *Cassia timoriensis* was positively correlated to its high phenolic and flavonoids. Phenolic compounds with anti-AChE activity were suggested to improve the signal transmission in nerve synapses and increase the concentration of ACh in synapses between cholinergic neurons [39,40]. In fact, rutin, quercetin, and kaempferol, were proven as cholinesterase inhibitors [41]. Furthermore, in silico studies revealed that compounds, such as rutin [42] and isorhamnetin [43] effectively inhibited the tyrosinase enzyme. The results of α- amylase and α-glucosidase inhibition might explain, in part, and support previous in vivo studies on the antidiabetic activity of the leaves of *C. fistula* [44] and *S. alexandrina* [45], as well as an in vitro study on the *S. italica* leaf [46]. In addition, the inhibition effect of the leaf of *C. grandis* shown in the present study, as well as previous in vivo studies demonstrating the hypoglycemic effect of the stem bark [16] and fruit pulp [37], suggest that all parts of this plant merit an in depth investigation on its antidiabetic activity. Furthermore, compounds, such as quercetin, ellagic acid, and kaempferol were found to possess a significant α-glucosidase inhibition effect [47,48,49].

### 2.5. Data Mining

**Figure 1:**
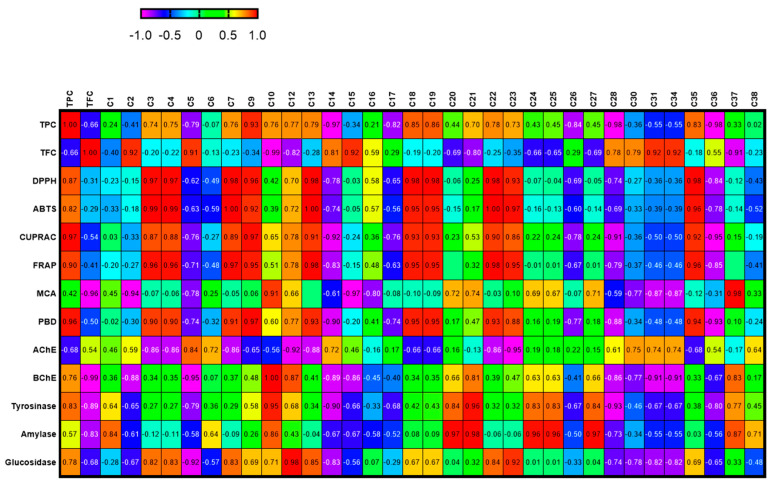
Relationship between secondary metabolites and biological activities of the tested extracts. For compound numbers refer to Table 1. The correlation is considered positive and statistically significant (r > 0.7).

**Figure 2:**
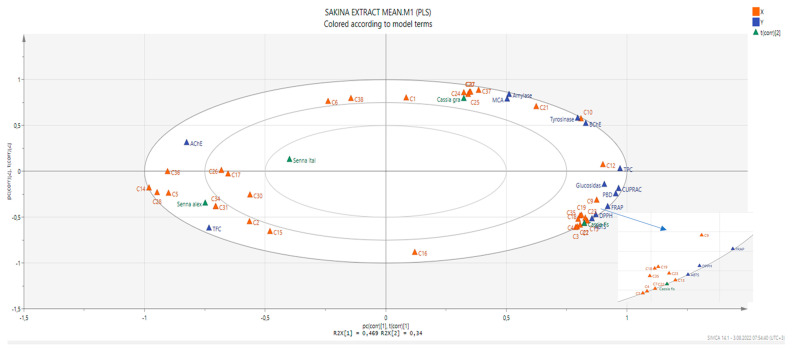
The biplot obtained from partial least squares regression describing the relationship between chemical compounds and bioactivities. For compound numbers refer to Table 1.

In order to establish a connection between the chemical components and biological effects of the tested extracts, we performed a multivariate statistical analysis. Recently, multivariate statistical approaches have gained interest to identify relationships between different parameters. This gives a good insight into the structure–activity relationship in phytochemical studies. To this end, we first performed a Pearson’s correlation analysis between bioactive components (as total and individually) and biological properties. As can be seen in Figure 1, the total phenolic content was highly correlated with the radical scavenger assay, reducing power assay, and three enzyme inhibition assays (BChE, tyrosinase, and glucosidase). In particular, based on the existence of a strong correlation between the radical scavenger and reducing power assays, we concluded that the same components might play a key role in the assays. This fact was also confirmed by the correlation values between individual phenolics (especially C7 (epicatechin), C9 (caffeic acid), C13 (procyanidin A2), C18 (isoquercitrin), and C19 (delphindin 3,5 diglucoside)) and these parameters. Consistent with our results, several researchers reported that there is a positive correlation between these parameters [50,51,52]. Concerning enzyme inhibitory assays, different results were observed. For example, for AChE, C5 (chlorogenic acid) might be the main contributor with a high correlation value (R > 0.8). As for BChE, C10 (vanillic acid) and C12 (syringic acid) were the main contributors. In addition, the observed tyrosinase inhibitory effect can be attributed to the presence of C10 and C21 (naringin). PLS-DA makes it possible to assess the relationship between the tested parameters and species. The tested plants were separated based on genus and two groups were observed (Figure 2). In general, the *Cassia* species were characterized by stronger antioxidant properties and a higher content of phenols. However, the *Senna* species were close and characterized by high flavonoid content, and they were richer in rutin, quercetin, and isorhamnetin.

### 2.6. Molecular Modeling

**Figure 3:**
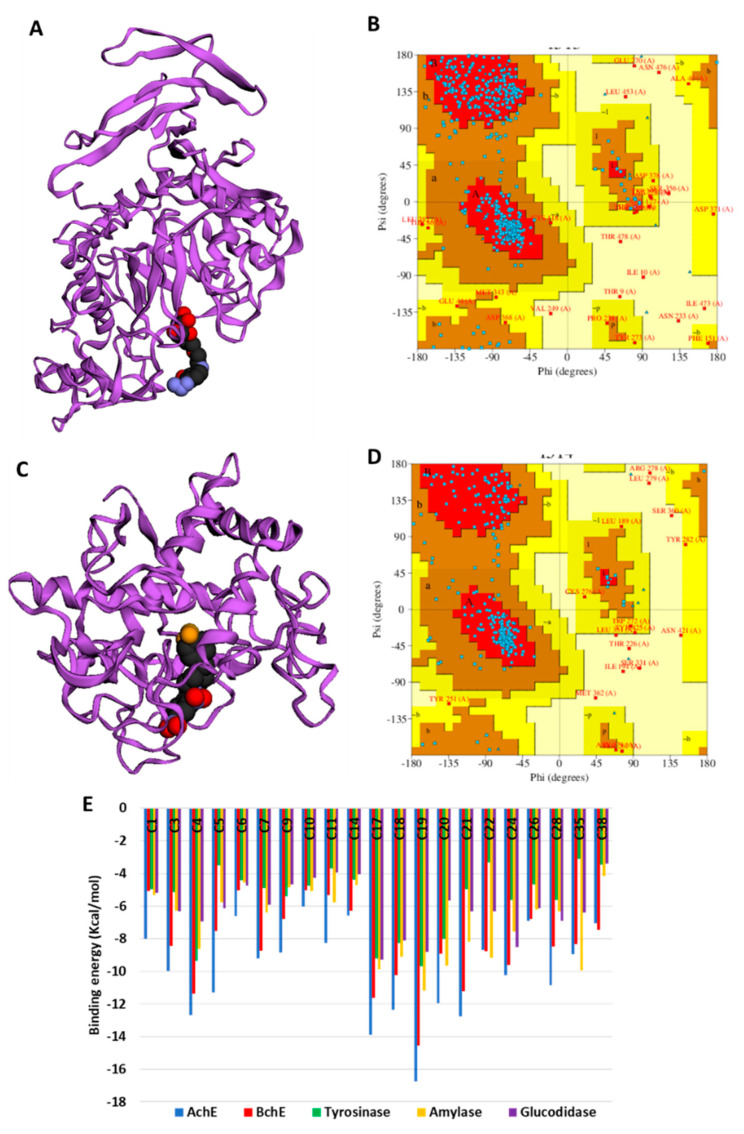
(**A**) Homology modeled structures of glucosidase, and (**B**) its Ramachandran plot showing the energetically allowed regions. (**C**) Homology modeled structures of tyrosinase and (**D**) its Ramachandran plot showing the energetically allowed regions. Binding energy (docking score) values of the top bioactive compounds from two Cassia and two Senna species. The compounds are abbreviated based on their serial number in Table 2.

**Figure 4:**
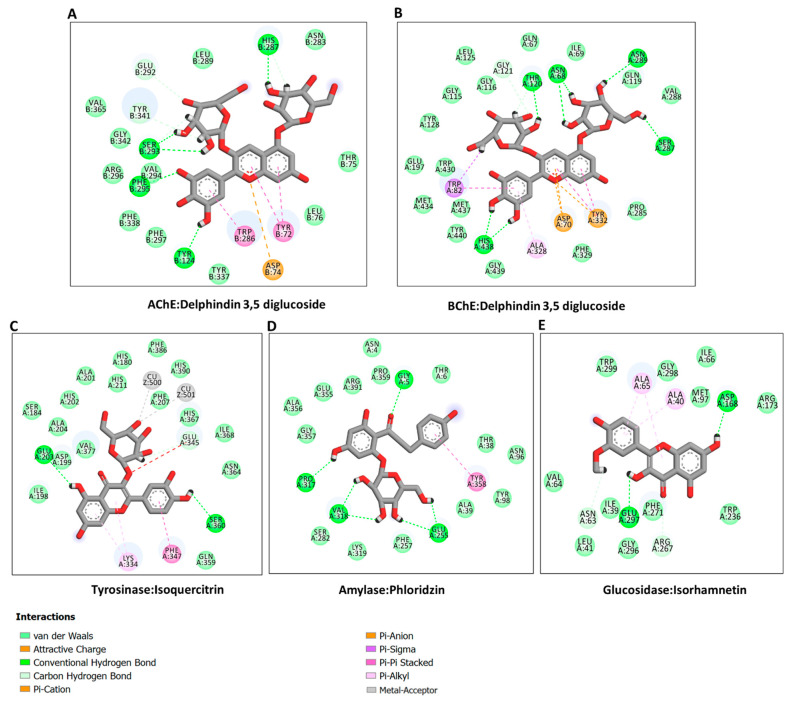
Protein–ligand interactions: (**A**) ChE:delphindin 3,5 diglucoside; (**B**) BChE:delphindin 3,5 diglucoside; (**C**) tyrosinase: isoquercitrin; (**D**) amylase: phloridzin; and (**E**) glucosidase:isorhamnetin.

In the absence of the crystal structures of human glucosidase and tyrosinase, homology models were built, as described in Section 3. The homology modeled structures of glucosidase and tyrosinase and their respective Ramachandran plot showing the energetically allowed regions of the 3D structures are shown in Figure 3A–D. The docking scores of the bioactive compounds in two *Cassia* and two *Senna* species against the five enzymes are shown in Figure 3E. Overall, the compounds showed the strongest binding to AChE and BChE, and moderate binding to tyrosinase, amylase, and glucosidase. Delphindin 3,5 diglucoside (C19) demonstrated the tightest binding to both AChE and BChE compared with the rest of the enzymes. Hence, the detailed protein–ligand interactions were visualized for some selected compounds. Both enzymes formed multiple H-bonds, a couple of charged interactions, and several van der Waals interactions (Figure 4A,B). On the other hand, isoquercitrin formed a metal–acceptor interaction with the two copper metal ions deep in the catalytic channel of tyrosinase. In addition, two H-bonds and two π-alkyl interactions near the active site surface, a π-π stacked, and several van der Waals interactions formed, contributing to the binding (Figure 4C). In the case of amylase, the major interactions formed by the active site residues with phloridzin are multiple H-bonds and several van der Waals interactions all over the channel (Figure 4D), while isorhamnetin, being a smaller molecule, spanned the glucosidase catalytic channel, forming H-bonds, alkyl, and multiple van der Waals interactions (Figure 4E). Together, these interactions may allow the compounds to block the activities of the studied enzymes.

**Figure 5:**
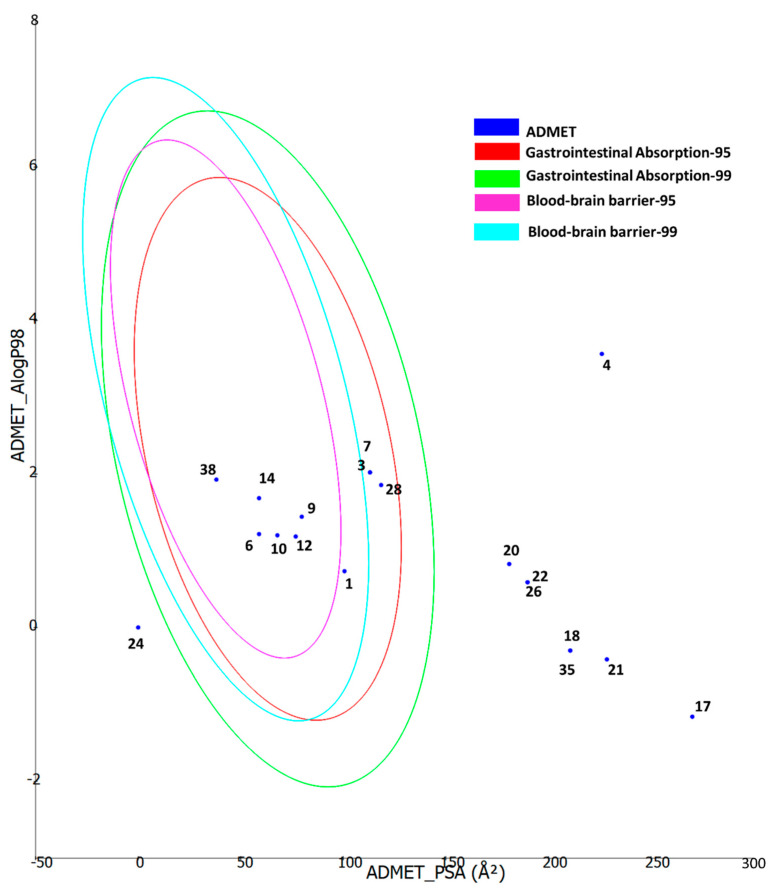
ADMET properties of the bioactive compounds extracted from two Cassia and two Senna species predicted using the Biovia DS ADMET prediction toolkit. The four ellipses enclose the area where well-absorbed compounds should be found: at 95 and 99% confidence levels for gastrointestinal absorption (red and green), and for blood–brain barrier penetration (magenta and aqua). The compounds are shown according to their serial number in Table 2.

Furthermore, the ADMET properties of the bioactive compounds were predicted using the Biovia DS ADMET prediction toolkit. The four ellipses enclose the area where well-absorbed compounds should be found: at 95 and 99% confidence levels for gastrointestinal absorption (red and green), and for the blood–brain barrier penetration (magenta and aqua). The compounds are shown according to their serial number (Figure 5). Compounds with low molecular weight and low polarity were found to be in one or more ellipses, suggesting low absorption and low blood–brain barrier penetration probability, whereas those with high molecular weight and high polarity were found to be located outside the ellipses, suggesting low absorption and low blood–brain barrier penetration probability.

## 3. Materials and Methods

### 3.1. Plant Materials

Fresh leaves of *C. fistula, C. grandis, S. alexandrina*, and *S. italica* were harvested from plants growing wild in the Botanical Garden in Al Mogran, Khartoum State, situated at 15°30′2″ N 32°33′36″ E. Taxonomical identification was carried out by Prof. Maha Kordofani and voucher specimens (No. HCF/05/19 for *C. fistula*, HCG/05/19 for *C. grandis*, HSA/05/19 for *S. alexandrina*, and HSI/05/19 for *S. italica*) were deposited at the Herbarium of the department.

### 3.2. Preparation of Extracts

Separately, 20 g of dried powdered leaves of each species were extracted by maceration in methanol (400 mL each) using a shaker apparatus, for about 24 h at room temperature, filtered, and then the solvent was evaporated under vacuum using a rotary evaporator. The resultant dry extracts were weighed and stored at 4 °C until used.

### 3.3. Determination of Total Polyphenol and Flavonoid Contents

The total phenolic and flavonoid contents were determined using the Folin–Ciocâlteu and AlCl_3_ tests, respectively [53]. The results were presented as gallic acid equivalents (mg GAEs/g dry extract) and rutin equivalents (mg REs/g dry extract) for the assays.

### 3.4. Phytochemical Analysis by HPLC–MS/MS System

The quantities of 38 phenolic compounds from the chemical classes of phenolic acids, flavonols, flavan-3-ols, flavones, proanthocyanidins, anthocyanins, and non-phenolic acids in the examined plants were measured using the methodology we previously described [54]. The dried extracts were dissolved in methanol (5 mg/mL), sonicated for 2 min at room temperature, and samples were centrifuged for 10 min at 13,000 rpm. Prior to being injected into the HPLC–MS/MS system, the solutions were filtered using a 0.2 m syringeless filter. Using an Agilent 1290 Infinity series and an Agilent Technology (Santa Clara, CA) Triple Quadrupole 6420 equipped with an electrospray ionization (ESI) source that operates in both positive and negative ionization modes, HPLC–MS/MS studies were conducted. The MS/MS parameters of each analyte were adjusted in flow injection analysis using Optimizer Software (Agilent). The target compounds were separated using Phenomenex’s Synergi Polar-RP C18 analytical column (250 mm × 4.6 mm, 4 μm), which was preceded by a Polar RP security guard cartridge (4 mm × 3 mm ID). The mobile phase used in gradient elution mode consisted of (A) water and (B) methanol, both contained a percentage of formic acid, and flowed at a rate of 0.8 mL min^−1^. The composition of the mobile phase was altered as follows: 0–1 min, isocratic condition, 20% B; 1–25 min, 20–85% B; 25–26 min, isocratic condition, 85% B; 26–32 min, 85–20% B. A total injection of 2 μL of the extracts was made and the column temperature was fixed at 30 °C. The ionization source’s drying gas temperature was maintained at 350 °C. The nebulizer pressure was 55 psi, the capillary voltage was 4000 V, and the gas flow rate was 12,000 mL/min. The areas of peaks of the most abundant product ions were integrated for quantification after the analytes were detected using the dynamic multiple reaction monitoring mode. Table S1 (Supplementary Materials) reported the HPLC–MS/MS acquisition parameters (dynamic MRM mode) used for the analysis of the 38 marker compounds.

### 3.5. Antioxidant Assays

Antioxidant assays were performed using methods that have been previously reported [55,56]. The antioxidant potential was calculated as follows: mg Trolox equivalents (TE)/g extract in the 2,2-diphenyl-1-picrylhydrazyl (DPPH) and 2,2’-azino-bis(3-ethylbenzothiazoline-6-sulfonic acid) (ABTS) radical scavenging tests; cupric reducing antioxidant capacity (CUPRAC) and ferric reducing antioxidant power (FRAP); mmol TE/g extract in the phosphomolybdenum (PBD) assays and mg disodium edetate equivalents (EDTAE)/g in the metal chelating activity (MCA).

### 3.6. Enzyme Inhibitory Assays

The enzyme inhibition experiments were performed based on previously described procedures [55,56]. Amylase and glucosidase inhibition was expressed as mmol acarbose equivalents (ACAE)/g extract, while acetylcholinesterase (AChE) and butyrylcholinesterase (BChE) inhibition was expressed as mg galanthamine equivalents (GALAE)/g extract. Tyrosinase inhibition was expressed as mg kojic acid equivalents (KAE)/g extract.

### 3.7. Molecular Modeling

The following crystal structures of the target enzymes were retrieved from the protein data bank (https://www.rcsb.org/ (accessed on 1 June 2022)): AChE (PDB ID: 6O52) [57], BChE (PDB ID: 6EQP) [58], tyrosinase (PDB ID: 6QXD) [59], amylase (PDB ID: 2QMK) [60], and glucosidase (PDB ID: 7KBJ) [61]. In the absence of crystal structures of human tyrosinase and glucosidase, human sequences (UniProt IDs P14679 and P0DUB6, respectively) were used to build their homology models using these PDB structures as templates. The models were built using the ITASSER web-based tool (https://zhanggroup.org/I-TASSER/, accessed on 1 June 2022) [62] and validated using the PROCHECK server (https://www.ebi.ac.uk/thornton-srv/software/PROCHECK/, accessed on 1 June 2022) [63].

Each protein was protonated using the predicted pKa of the titratable residues at a physiological pH of 7.4 using an online server “Playmolecule proteinPrepare” (https://playmolecule.com/proteinPrepare/, accessed on 1 June 2022) [64]. The three-dimensional structure of each study ligand was retrieved from the PubChem database (https://pubchem.ncbi.nlm.nih.gov/, accessed on 1 June 2022), and its geometry was optimized using Frog2 [65].

Using Autodock Tools program (https://autodock.scripts.edu, accessed on 10 June 2022) [66], docking grid files were generated using the coordinates of the cocrystal ligand in each crystal. The details of the docking procedure have been described in our previous studies [67,68]. The binding energy of the ligand poses were calculated, and protein–ligand interactions were examined using the Biovia DS Visualizer (Dassault Systèmes Biovia Software Inc, 2012, San Diego, CA, USA).

### 3.8. Data Analysis

All analyses were done in triplicate and the results were given as means ± SD. Pearson’s correlation coefficients were calculated to analyze the relationship between secondary metabolites, and antioxidant, and enzyme inhibitory activities, respectively. Pearson’s correlation was performed by GraphPad version 9.0. The relationship between species, chemical compounds, and bioactivities was also assessed using partial least squares regression analysis (PLS-DA). PLS-DA analysis was performed by SIMCA version 14.0.

## 4. Conclusions

The leaf methanolic extracts of the *Cassia* and *Senna* species were rich in phenols and the two species of the former genus contained higher amounts of total polyphenolics content, while those from the latter had a higher total flavonoid content. *C. fistula* and *C. grandis* were dominated by epicatechin and kaempferol-3-glucoside, respectively, while rutin was the major compound in both *Senna* spp. All four species showed considerable antioxidant activity, and *C. fistula* exerted the highest activity in most assays. Furthermore, the four species showed significant enzyme inhibition capacity. The outcome of the present study is helpful in the identification of new bioactive compounds from the investigated species, which might be further explored in the development of an effective drug system for the treatment of many diseases. Furthermore, more bioactive compounds belonging to different classes of secondary metabolites should be identified to better understand the mechanisms underlying the pharmacological benefits of these extracts.

## Figures and Tables

**Table 1:** Total polyphenolic and flavonoid contents of leaf methanolic extracts of the *Cassia* and *Senna* species.

Plant Name	TPC (mg GAE/g)	TFC (mg RE/g)
*Cassia fistula*	49.75 ± 0.37 ^a^	39.15 ± 0.29 ^c^
*Cassia grandis*	42.23 ± 0.27 ^b^	30.38 ± 0.22 ^d^
*Senna alexandrina*	22.25 ± 0.29 ^c^	59.24 ± 0.08 ^a^
*Senna italica*	21.54 ± 0.28 ^c^	41.47 ± 0.28 ^b^

Values are reported as mean ± SD. TPC: total phenolic content; TFC: total flavonoid content; GAE: gallic acid equivalent; RE: rutin equivalent; TE: Trolox equivalent. Different letters in the same column indicate significant differences in the samples (*p* < 0.05).

**Table 2:** Content (µg g^−1^ of dried extract) of bioactive compounds in leaf methanolic extracts of the *Cassia* and *Senna* species.

No	Compound	*C. fistula*	*C. grandis*	*S. alexandrina*	*S. italica*
1	Gallic acid	37.80	88.03	53.98	37.53
2	Neochlorogenic acid	0.15	n.d.	0.69	n.d.
3	Catechin	1361.85	32.94	3.19	n.d.
4	Procyanidin B2	1767.78	62.43	n.d.	17.20
5	Chlorogenic acid	2.29	3.55	11.45	5.54
6	4-Hydroxy benzoic acid	200.01	466.62	364.13	258.74
7	Epicatechin	8928.75	492.80	5.72	n.d.
8	3-Hydroxybenzoic acid	n.d.	n.d.	n.d.	n.d.
9	Caffeic acid	101.65	76.69	63.16	48.40
10	Vanillic acid	179.59	207.39	102.62	155.01
11	Resveratrol	n.d.	n.d.	n.d.	n.d.
12	Syringic acid	112.42	85.02	24.59	78.69
13	Procyanidin A2	35.90	7.34	3.63	4.60
14	P-Coumaric acid	177.89	198.16	354.55	314.18
15	Ferulic acid	21.43	5.52	49.04	7.73
16	3,5-Dicaffeoylquinic acid	0.67	n.d.	0.61	n.d.
17	Rutin	684.39	2.89	6381.85	17,285.02
18	Isoquercitrin	6526.84	3169.68	2752.63	1042.35
19	Delphindin 3,5 diglucoside	4907.12	2423.86	2071.99	810.07
20	Phloridzin	101.26	2106.51	0.56	10.85
21	Naringin	163.84	444.87	n.d.	16.14
22	Quercitrin	1563.25	139.76	5.30	2.82
23	Myricetin	4.26	1.28	0.71	1.56
24	Kaempferol-3-glucoside	3398.12	47,360.04	3019.95	1082.75
25	Ellagic acid	10.62	116.46	8.61	n.d.
26	Quercetin	106.15	126.86	522.83	1207.88
27	Phloretin	0.89	16.65	0.05	n.d.
28	Isorhamnetin	7.83	6.92	454.47	411.96
29	Delphindin3-galactoside	n.d.	n.d.	n.d.	n.d.
30	Cyanidin-3-glucoside	18.27	24.23	56.93	11.18
31	Petunidin-3-glucoside	n.d.	n.d.	2.51	n.d.
32	Pelargonidin-3-rutinoside	n.d.	n.d.	n.d.	n.d.
33	Pelargonidin-3-glucoside	n.d.	n.d.	n.d.	n.d.
34	Malvidin-3-galactoside	n.d.	n.d.	14.19	n.d.
35	Hyperoside	8270.74	3634.40	3299.95	1293.52
36	Hesperidin	n.d.	12.34	56.04	74.06
37	Kaempferol	28.04	49.89	16.81	37.98
38	Trans-cinnamic acid	177.68	3851.66	2095.98	803.88
Total content		38,897.47	65,214.79	21,798.71	25,019.64

n.d., not detected.

**Table 3:** Antioxidant activity of leaf methanolic extracts of the *Cassia* and *Senna* species.

Plant name	DPPH (mg TE */g)	ABTS (mg TE/g)	CUPRAC (mg TE/g)	FRAP (mg TE/g)	MCA (mg EDTAE **/g)	PBD (mmol TE/g)
*Cassia fistula*	77.36 ± 0.69 ^a^	218.44 ± 13.8 ^a^	215.33 ± 4.37 ^a^	106.34 ± 5.88 ^a^	5,68 ± 0.55 ^c^	1.94 ± 0.13 ^a^
*Cassia grandis*	34.74 ± 0.26 ^b^	78.43 ± 0.08 ^b^	152.79 ± 2.19 ^b^	62.68 ± 0.24 ^b^	11.38 ± 0.96 ^a^	1.48 ± 0.12 ^b^
*Senna alexandrina*	24.74 ± 0.64 ^c^	54.49 ± 1.28 ^c^	88.45 ± 2.11 ^c^	44.02 ± 0.92 ^c^	na	1.11 ± 0.05 ^c^
*Senna italica*	18.60 ± 0.20 ^d^	51.98± 0.05 ^c^	84.60 ± 2.54 ^c^	44.58 ± 0.26 ^c^	8.11 ± 0.72 ^b^	1.07 ± 0.11 ^c^

Note: Values are reported as mean ± SD. DPPH: 2,2-diphenyl-1-picrylhydrazyl; ABTS: 2,2’-azino-bis(3-ethylbenzothiazoline-6-sulfonic acid); CUPRAC: cupric ion reducing antioxidant capacity; FRAP: ferric reducing antioxidant power; MCA: metal chelating activity; PBD: phosphomolybdenum. * TE, Trolox equivalents. ** EDTAE, disodium edetate equivalents. Different superscript letters in the same column indicate significant difference (*p* < 0.05).

**Table 4:** Enzyme inhibitory activity of leaf methanolic extracts of the *Cassia* and *Senna* species.

Plant name	AChE (mg GALAE */g)	BChE (mg GALAE/g)	Tyrosinase (mg KAE **/g)	Amylase (mmol ACAE ***/g)	Glucosidase (mmol ACAE/g)
*Cassia fistula*	1.71 ± 0.20 ^b^	0.92 ± 0.02 ^a^	34.51 ± 0.93 ^b^	0.40 ± 0.02 ^b^	1.45 ± 0.01 ^a^
*Cassia grandis*	2.17 ± 0.22 ^a^	1.15 ± 0.18 ^a^	46.58 ± 0.32 ^a^	0.48 ± 0.02 ^a^	1.44 ± 0.00 ^ab^
*Senna alexandrina*	2.41 ± 0.07 ^a^	na	15.12 ± 0.59 ^d^	0.37 ± 0.02 ^b^	1.43 ± 0.00 ^b^
*Senna italica*	2.05 ± 0.11 ^ab^	0.64 ± 0.02 ^b^	21.17 ± 0.38 ^c^	0.39 ± 0.01 ^b^	1.44 ± 0.01 ^ab^

Note: Values are reported as mean ± SD. * GALAE, galanthamine equivalents. ** KAE, kojic acid equivalents. *** ACAE, acarbose equivalents. Different superscript letters in the same column indicate significant difference (*p* < 0.05); na, not active.

## Data Availability

Not applicable.

## Supplementary Materials

The following supporting information can be downloaded at: https://www.mdpi.com/article/10.3390/molecules27175590/s1, Table S1. HPLC–MS/MS acquisition parameters (dynamic-MRM mode) used for the analysis of the 38 marker compounds.

## References

[1] Lautié E., Russo O., Ducrot P., Boutin J.A. (2020). Unraveling plant natural chemical diversity for drug discovery purposes. Front. Pharmacol..

[2] Al-Nemi R., Makki A.A., Sawalha K., Hajjar D., Jaremko M. (2022). Untargeted Metabolomic Profiling and Antioxidant Capacities of Different Solvent Crude Extracts of Ephedra foeminea. Metabolites.

[3] Newman D.J., Cragg G.M. (2016). Natural products as sources of new drugs from 1981 to 2014. J. Nat. Prod..

[4] Veiga M., Costa E.M., Silva S., Pintado M. (2020). Impact of plant extracts upon human health: A review. Crit. Rev. Food Sci. Nutr..

[5] Iqbal A., Sher A.A., Muhammad N., Badshah S.L., Emwas A.-H., Jaremko M. (2022). Extraction and Fractionation of Prokinetic Phytochemicals from Chrozophora tinctoria and Their Bioactivities. Molecules.

[6] Lavanya B., Maheswaran A., Vimal N., Vignesh K., Uvarani K., Varsha R. (2018). An overall view of cassia species phytochemical constituents and its pharmacological uses. Int. J. Pharm. Sci. Res..

[7] Khurm M., Wang X., Zhang H., Hussain S.N., Qaisar M.N., Hayat K., Saqib F., Zhang X., Zhan G., Guo Z. (2021). The genus Cassia, L.: Ethnopharmacological and phytochemical overview. Phytother. Res..

[8] do Nascimento M.N.G., Martins M.M., Cunha L.C.S., de Souza Santos P., Goulart L.R., de Souza Silva T., Martins C.H.G., de Morais S.A.L., Pivatto M. (2020). Antimicrobial and cytotoxic activities of Senna and Cassia species (Fabaceae) extracts. Ind. Crops Prod..

[9] de Albuquerque Melo G.M., Silva M.C.R., Guimaraes T.P., Pinheiro K.M., da Matta C.B.B., de Queiroz A.C., Pivatto M., da Silva Bolzani V., Alexandre-Moreira M.S., Viegas C. (2014). Leishmanicidal activity of the crude extract, fractions and major piperidine alkaloids from the flowers of Senna spectabilis. Phytomedicine.

[10] Selegato D.M., Monteiro A.F., Vieira N.C., Cardoso P., Pavani V.D., Bolzani V.S., Castro-Gamboa I. (2017). Update: Biological and chemical aspects of Senna spectabilis. J. Braz. Chem. Soc..

[11] Sanoria S., Qadrie Z.L., Gautam S.P., Barwal A. (2020). Cassia Fistula: Botany, phytochemistry and pharmacological leverages—A review. Int. J. Pharm. Pharm. Sci..

[12] Abdalla W.E., Guma’a A.G.N., El Ghazali G.E.B., Khalid H.E.S. (2016). An Updated Species Check-list for the Genus Cassia L. sensu lato in the Sudan. Nat. Resour. Res..

[13] Yagi S., Yagi A. (2021). Important Medicinal Plants—Sudan, in Medicinal and Aromatic Plants of the World, in Encyclopedia of Life Support Systems (EOLSS).

[14] Musa S.M., Fathelrhman E.A., Elsheikh A.E., Lubna A.M.N.A., Abdel L.E.M., Sakina M.Y. (2011). Ethnobotanical study of medicinal plants in the Blue Nile State, South-eastern Sudan. J. Med. Plants Res..

[15] Anis M., Siddique I., Naz R., Ahmed M.R., Aref I.M., Bandani A.R. (2012). Advances in Micropropagation of a Highly Important Cassia species—A Review. New Perspectives in Plant Protection.

[16] Lodha S.R., Joshi S.V., Vyas B.A., Upadhye M.C., Kirve M.S., Salunke S.S., Kadu S.K., Rogye M.V. (2010). Assessment of the antidiabetic potential of Cassia grandis using an in vivo model. J. Adv. Pharm. Technol. Res..

[17] Yang M., Ma Y., Wang Z., Khan A., Zhou W., Zhao T., Cao J., Cheng G., Cai S. (2020). Phenolic constituents, antioxidant and cytoprotective activities of crude extract and fractions from cultivated artichoke inflorescence. Ind. Crops Prod..

[18] Tzekiat L., Chiang L.K. (2013). Total phenolics, total tannins and antioxidant activity of Cassia fistula L. extracts of bark, stem, leaf and root under different age classes. Asian J. Pharm. Res. Health Care.

[19] Fuentes J.A.M., Fernández I.M., Fernández H.Z., Sánchez J.L., Alemán R.S., Navarro-Alarcon M., Borrás-Linares I., Maldonado S.A.S. (2020). Quantification of bioactive molecules, minerals and bromatological analysis in Carao (Cassia grandis). J. Agric. Sci..

[20] Laghari A.Q., Memon S., Nelofar A., Laghari A.H. (2011). Extraction, identification and antioxidative properties of the flavonoid-rich fractions from leaves and flowers of Cassia angustifolia. Am. J. Anal. Chem..

[21] Gololo S.S., Mapfumari N.S., Mogale M.A. (2018). Comparative qualitative phytochemical analysis of the leaves of sena italica collected from different areas in Limpopo Province, South Africa. Int. J. Pharm. Pharm. Sci..

[22] Gritsanapan W., Tantisewie B., Jirawongse V. (1984). Chemical-Constituents of Cassia-Timorensis and Cassia-Grandis. J. Sci. Soc. Thail..

[23] Verma R.P., Sinha K.S. (1996). Anthraquinone ß-D-glucoside from Cassia grandis. Int. J. Pharmacogn..

[24] Kalidhar M.S.B. (1998). Chemical Examination of the Stems of *Cassia grandis* L. Indian J. Pharm. Sci..

[25] Acidri R., Sawai Y., Sugimoto Y., Handa T., Sasagawa D., Masunaga T., Yamamoto S., Nishihara E. (2020). Phytochemical profile and antioxidant capacity of coffee plant organs compared to green and roasted coffee beans. Antioxidants.

[26] Al-Haidari R.A., Al-Oqail M.M. (2020). New benzoic acid derivatives from Cassia italica growing in Saudi Arabia and their antioxidant activity. Saudi Pharm. J..

[27] Fernández-Poyatos M.d.P., Zengin G., Salazar-Mendías C., Ruiz-Medina A., Sinan K.I., Llorent-Martínez E.J. (2020). Study on Three Sarcocapnos Species as Potential Sources of Bioactive Compounds: Relation between Phenolic Content and Bioactivity by Multivariate Analysis. J. Anal. Methods Chem..

[28] Ismail B.B., Pu Y., Guo M., Ma X., Liu D. (2019). LC-MS/QTOF identification of phytochemicals and the effects of solvents on phenolic constituents and antioxidant activity of baobab (*Adansonia digitata*) fruit pulp. Food Chem..

[29] Lee K.W., Kim Y.J., Lee H.J., Lee C.Y. (2003). Cocoa has more phenolic phytochemicals and a higher antioxidant capacity than teas and red wine. J. Agric. Food Chem..

[30] Gülçin İ. (2006). Antioxidant activity of caffeic acid (3, 4-dihydroxycinnamic acid). Toxicology.

[31] Calderon-Montano J.M., Burgos-Morón E., Pérez-Guerrero C., López-Lázaro M. (2011). A review on the dietary flavonoid kaempferol. Mini Rev. Med. Chem..

[32] Negro C., Longo L., Vasapollo G., Bellis L., Miceli A. (2012). Biochemical, antioxidant and anti-inflammatory properties of pomegranate fruits growing in Southern Italy (Salento, Apulia). Acta Aliment..

[33] Yagi S., Drouart N., Bourgaud F., Henry M., Chapleur Y., Laurain-Mattar D. (2013). Antioxidant and antiglycation properties of Hydnora johannis roots. S. Afr. J. Bot..

[34] Das A.B., Goud V.V., Das C. (2019). 9-Phenolic compounds as functional ingredients in beverages. Value-Added Ingredients and Enrichments of Beverages.

[35] Gao Y., Fang L., Wang X., Lan R., Wang M., Du G., Guan W., Liu J., Brennan M., Guo H. (2019). Antioxidant activity evaluation of dietary flavonoid hyperoside using saccharomyces cerevisiae as a model. Molecules.

[36] Orhan I.E. (2019). Enzyme inhibitors as the attractive targets for the treatment of various diseases. Curr. Med. Chem..

[37] Prada A.L., Amado J.R.R., Keita H., Zapata E.P., Carvalho H., Lima E.S., de Sousa T.P., Carvalho J.C.T. (2018). Cassia grandis fruit extract reduces the blood glucose level in alloxan-induced diabetic rats. Biomed. Pharm..

[38] Alhawarri M.B., Dianita R., Razak K.N.A., Mohamad S., Nogawa T., Wahab H.A. (2021). Antioxidant, Anti-Inflammatory, and Inhibition of Acetylcholinesterase Potentials of Cassia timoriensis DC. Flowers. Molecules.

[39] Jabir N.R., Khan F.R., Tabrez S. (2018). Cholinesterase targeting by polyphenols: A therapeutic approach for the treatment of Alzheimer’s disease. CNS Neurosci. Ther..

[40] Szwajgier D. (2015). Anticholinesterase activity of selected phenolic acids and flavonoids-interaction testing in model solutions. Ann. Agric. Environ. Med..

[41] Szwajgier D., Borowiec K., Zapp J. (2020). Activity-guided isolation of cholinesterase inhibitors quercetin, rutin and kaempferol from *Prunus persica* fruit. Z. Für Nat. C.

[42] Si Y.-X., Yin S.-J., Oh S., Wang Z.-J., Ye S., Yan L., Yang J.-M., Park Y.-D., Lee J., Qian G.-Y. (2012). An integrated study of tyrosinase inhibition by rutin: Progress using a computational simulation. J. Biomol. Struct. Dyn..

[43] Si Y.-X., Wang Z.-J., Park D., Jeong H.O., Ye S., Chung H.Y., Yang J.-M., Yin S.-J., Qian G.-Y. (2012). Effects of isorhamnetin on tyrosinase: Inhibition kinetics and computational simulation. Biosci. Biotechnol. Biochem..

[44] Einstein J.W., Mohd Rais M., Mohd M.A. (2013). Comparative Evaluation of the Antidiabetic Effects of Different Parts of *Cassia fistula* Linn, a Southeast Asian Plant. J. Chem..

[45] Jani D.K., Goswami S. (2020). Antidiabetic activity of Cassia angustifolia Vahl. and Raphanus sativus Linn. leaf extracts. J. Tradit. Complement. Med..

[46] Malematja R.O., Bagla V.P., Njanje I., Mbazima V., Poopedi K.W., Mampuru L., Mokgotho M.P. (2018). Potential Hypoglycaemic and Antiobesity Effects of *Senna italica* Leaf Acetone Extract. Evid.-Based Complement. Altern. Med..

[47] Praparatana R., Maliyam P., Barrows L.R., Puttarak P. (2022). Flavonoids and Phenols, the Potential Anti-Diabetic Compounds from Bauhinia strychnifolia Craib. Stem. Molecules.

[48] Qi Y. (2012). Anti-diabetic activities of phenolic compounds in muscadine against alpha-glucosidase and pancreatic lipase. LWT—Food Sci. Technol..

[49] Sarian M.N., Ahmed Q.U., Mat So’ad S.Z., Alhassan A.M., Murugesu S., Perumal V., Syed Mohamad S.N.A., Khatib A., Latip J. (2017). Antioxidant and Antidiabetic Effects of Flavonoids: A Structure-Activity Relationship Based Study. BioMed Res. Int..

[50] Wairata J., Fadlan A., Setyo Purnomo A., Taher M., Ersam T. (2022). Total phenolic and flavonoid contents, antioxidant, antidiabetic and antiplasmodial activities of Garcinia forbesii King: A correlation study. Arab. J. Chem..

[51] Halim M.A., Kanan K.A., Nahar T., Rahman M.J., Ahmed K.S., Hossain H., Mozumder N.H.M.R., Ahmed M. (2022). Metabolic profiling of phenolics of the extracts from the various parts of blackberry plant (*Syzygium cumini* L.) and their antioxidant activities. LWT.

[52] Lin Y., Tang D., Liu X., Cheng J., Wang X., Guo D., Zou J., Yang H. (2022). Phenolic profile and antioxidant activity of longan pulp of different cultivars from South China. LWT.

[53] Zengin G., Aktumsek A. (2014). Investigation of antioxidant potentials of solvent extracts from different anatomical parts of Asphodeline anatolica E. Tuzlaci: An endemic plant to Turkey. Afr. J. Tradit. Complement. Altern. Med..

[54] Mustafa A.M., Angeloni S., Abouelenein D., Acquaticci L., Xiao J., Sagratini G., Maggi F., Vittori S., Caprioli G. (2022). A new HPLC-MS/MS method for the simultaneous determination of 36 polyphenols in blueberry, strawberry and their commercial products and determination of antioxidant activity. Food Chem..

[55] Uysal S., Zengin G., Locatelli M., Bahadori M.B., Mocan A., Bellagamba G., De Luca E., Mollica A., Aktumsek A. (2017). Cytotoxic and enzyme inhibitory potential of two Potentilla species (*P. speciosa* L. and *P. reptans* Willd.) and their chemical composition. Front. Pharmacol..

[56] Grochowski D.M., Uysal S., Aktumsek A., Granica S., Zengin G., Ceylan R., Locatelli M., Tomczyk M. (2017). In vitro enzyme inhibitory properties, antioxidant activities, and phytochemical profile of Potentilla thuringiaca. Phytochem. Lett..

[57] Gerlits O., Ho K.-Y., Cheng X., Blumenthal D., Taylor P., Kovalevsky A., Radić Z. (2019). A new crystal form of human acetylcholinesterase for exploratory room-temperature crystallography studies. Chem.-Biol. Interact..

[58] Rosenberry T.L., Brazzolotto X., Macdonald I.R., Wandhammer M., Trovaslet-Leroy M., Darvesh S., Nachon F. (2017). Comparison of the Binding of Reversible Inhibitors to Human Butyrylcholinesterase and Acetylcholinesterase: A Crystallographic, Kinetic and Calorimetric Study. Molecules.

[59] Ielo L., Deri B., Germanò M.P., Vittorio S., Mirabile S., Gitto R., Rapisarda A., Ronsisvalle S., Floris S., Pazy Y. (2019). Exploiting the 1-(4-fluorobenzyl)piperazine fragment for the development of novel tyrosinase inhibitors as anti-melanogenic agents: Design, synthesis, structural insights and biological profile. Eur. J. Med. Chem..

[60] Maurus R., Begum A., Williams L.K., Fredriksen J.R., Zhang R., Withers S.G., Brayer G.D. (2008). Alternative Catalytic Anions Differentially Modulate Human α-Amylase Activity and Specificity. Biochemistry.

[61] Karade S.S., Hill M.L., Kiappes J.L., Manne R., Aakula B., Zitzmann N., Warfield K.L., Treston A.M., Mariuzza R.A. (2021). N-Substituted Valiolamine Derivatives as Potent Inhibitors of Endoplasmic Reticulum α-Glucosidases I and II with Antiviral Activity. J. Med. Chem..

[62] Yang J., Zhang Y. (2015). I-TASSER server: New development for protein structure and function predictions. Nucleic Acids Res..

[63] Laskowski R.A., MacArthur M.W., Moss D.S., Thornton J.M. (1993). PROCHECK: A program to check the stereochemical quality of protein structures. J. Appl. Crystallogr..

[64] Martínez-Rosell G., Giorgino T., De Fabritiis G. (2017). PlayMolecule ProteinPrepare: A Web Application for Protein Preparation for Molecular Dynamics Simulations. J. Chem. Inf. Modeling.

[65] Miteva M.A., Guyon F., Tuffery P. (2010). Frog2: Efficient 3D conformation ensemble generator for small compounds. Nucleic Acids Res..

[66] Morris G.M., Huey R., Lindstrom W., Sanner M.F., Belew R.K., Goodsell D.S., Olson A.J. (2009). AutoDock4 and AutoDockTools4: Automated docking with selective receptor flexibility. J. Comput. Chem..

[67] Kurt-Celep I., Zheleva-Dimitrova D., Gevrenova R., Uba A.I., Zengin G., Yıldıztugay E., Picot-Allain C.M.N., Lorenzo J.M., Mahomoodally M.F., Montesano D. (2022). An In-Depth Study on the Metabolite Profile and Biological Properties of Primula auriculata Extracts: A Fascinating Sparkle on the Way from Nature to Functional Applications. Antioxidants.

[68] Zengin G., Uba A.I., Ocal M., Sharifi-Rad M., Caprioli G., Angeloni S., Altunoglu Y.C., Baloglu M.C., Yıldıztugay E. (2022). Integration of in vitro and in silico approaches to assess three Astragalus species from Turkey flora: A novel spotlight from lab bench to functional applications. Food Biosci..

