# Supplementary material for: Phenolic Profile, Antioxidant and Enzyme Inhibitory Activities of Leaves from Two Cassia and Two Senna Species
Source: Molecules. 2022 Aug 30;27(17):5590. doi: 10.3390/molecules27175590 (PMC9457634; doi:10.3390/molecules27175590)
Supplement: Supplementary file 1 [file molecules-27-05590-s001.zip › molecules-1882765-supplementary.pdf]

molecules

supplementary material

Phenolic Profile and Antioxidant and Enzyme Inhibitory Activities of Leaves from Two *Cassia* and Two *Senna* Species

Haifa A. A. Omer [1,2], Giovanni Caprioli [3], Doaa Abouelenein [3], Ahmed M. Mustafa [3], Abdullahi Ibrahim Uba [4], Gunes Ak [5], Refiye Beyza Ozturk [5], Gokhan Zengin [5] and Sakina Yagi [2,*]

Assays for Total Phenolic and Flavonoid Contents

The total phenolic content was determined by employing the methods given in the literature with some modification. A sample solution (2 mg/ml, 0.25 mL) was mixed with diluted Folin–Ciocâlteu reagent (1 mL, 1:9, v/v) and shaken vigorously. After 3 min, a Na_2CO_3 solution (0.75 mL, 1%) was added and the sample absorbance was read at 760 nm after a 2 h incubation at room temperature. The total phenolic content was expressed as milligrams of gallic acid equivalents (mg GAE/g extract)(Uysal et al., 2017).

The total flavonoid content was determined using the $AlCl_3$ method. Briefly, a sample solution (2 mg/ml, 1 mL) was mixed with the same volume of aluminum trichloride (2%) in methanol. Similarly, a blank was prepared by adding a sample solution (1 mL) to methanol (1 mL) without $AlCl_3$. The sample and blank absorbances were read at 415 nm after a 10 min incubation at room temperature. The absorbance of the blank was subtracted from that of the sample. Rutin was used as a reference standard and the total flavonoid content was expressed as milligrams of rutin equivalents (mg RE/g extract) (Uysal et al., 2017).

Determination of Antioxidant and Enzyme Inhibitory Effects

Antioxidant (DPPH and ABTS radical scavenging, reducing power (CUPRAC and FRAP), phosphomolybdenum and metal chelating (ferrozine method)), and enzyme

inhibitory activities (cholinesterase (Elmann’s method), tyrosinase (dopachrome method), α-amylase (iodine/potassium iodide method), α -glucosidase (chromogenic PNPG method) and pancreatic lipase (*p*-nitrophenyl butyrate (p-NPB) method)) were determined using the methods previously described by Uysal et al. (Uysal et al., 2017) and Grochowski et al. (Grochowski et al., 2017)

For the DPPH (1,1-diphenyl-2-picrylhydrazyl) radical scavenging assay: A sample solution (0.2-2 mg/ml) was added to 4 mL of a 0.004% methanol solution of DPPH. The sample absorbance was read at 517 nm after a 30 min incubation at room temperature in the dark. DPPH radical scavenging activity was expressed as milligrams of Trolox equivalents (mg TE/g extract).

For the ABTS (2,2′-azino-bis(3-ethylbenzothiazoline) 6-sulfonic acid) radical scavenging assay: Briefly, ABTS+ was produced directly by reacting 7 mM ABTS solution with 2.45 mM potassium persulfate and allowing the mixture to stand for 12–16 min in the dark at room temperature. Prior to beginning the assay, the ABTS solution was diluted with methanol to an absorbance of 0.700 ± 0.02 at 734 nm. A sample solution (0.2-2 mg/ml) was added to the ABTS solution (2 mL) and mixed. The sample absorbance was read at 734 nm after a 30 min incubation at room temperature. The ABTS radical scavenging activity was expressed as milligrams of Trolox equivalents (mg TE/g extract).

For the CUPRAC (cupric ion reducing activity) activity assay: A sample solution (0.2-2 mg/ml) was added to a premixed reaction mixture containing $CuCl_2$ (1 mL, 10 mM), neocuproine (1 mL, 7.5 mM) and a NH_4Ac buffer (1 mL, 1 M, pH 7.0). Similarly, a blank was prepared by adding a sample solution (0.5 mL) to a premixed reaction mixture (3 mL)

without $CuCl_2$. Then, the sample and blank absorbances were read at 450 nm after a 30 min incubation at room temperature. The absorbance of the blank was subtracted from that of the sample. The CUPRAC activity was expressed as milligrams of Trolox equivalents (mg TE/g extract).

For the FRAP (ferric reducing antioxidant power) activity assay: A sample solution (0.2-2 mg/ml) was added to a premixed FRAP reagent (2 mL) containing acetate buffer (0.3 M, pH 3.6), 2,4,6-tris(2-pyridyl)-S-triazine (TPTZ) (10 mM) in 40 mM HCl, and ferric chloride (20 mM) in a ratio of 10:1:1 (v/v/v). Then, the sample absorbance was read at 593 nm after a 30 min incubation at room temperature. The FRAP activity was expressed as milligrams of Trolox equivalents (mg TE/g extract).

For the phosphomolybdenum method: A sample solution (0.2-2 mg/ml) was combined with 3 mL of reagent solution (0.6 M sulfuric acid, 28 mM sodium phosphate, and 4 mM ammonium molybdate). The sample absorbance was read at 695 nm after a 90 min incubation at 95 °C. The total antioxidant capacity was expressed as millimoles of Trolox equivalents (mmol TE/g extract).

For the metal chelating activity assay: Briefly, a sample solution (0.2-2 mg/ml) was added to the $FeCl_2$ solution (0.05 mL, 2 mM). The reaction was initiated by the addition of 5 mM ferrozine (0.2 mL). Similarly, a blank was prepared by adding a sample solution (2 mL) to the $FeCl_2$ solution (0.05 mL, 2 mM) and water (0.2 mL) without ferrozine. Then, the sample and blank absorbances were read at 562 nm after 10 min incubation at room temperature. The absorbance of the blank was subtracted from that of the sample. The metal

chelating activity was expressed as milligrams of EDTA (disodium edetate) equivalents (mg EDTAE/g extract).

For the Cholinesterase (ChE) inhibitory activity assay: A sample solution (0.2-2 mg/ml) was mixed with DTNB (5,5-dithio-bis(2-nitrobenzoic) acid, Sigma, St. Louis, MO, U.S.A.) (125 μL) and AChE (acetylcholines-terase (Electric ell acetylcholinesterase, Type-VI-S, EC 3.1.1.7, Sigma)), or BChE (butyrylcholinesterase (horse serum butyrylcholinesterase, EC 3.1.1.8, Sigma)) solution (25 μL) in Tris–HCl buffer (pH 8.0) in a 96-well microplate and incubated for 15 min at 25 °C. The reaction was then initiated with the addition of acetylthiocholine iodide (ATCI, Sigma) or butyrylthiocholine chloride (BTCl, Sigma) (25 μL). Similarly, a blank was prepared by adding a sample solution to all reaction reagents without the enzyme (AChE or BChE) solution. The sample and blank absorbances were read at 405 nm after 10 min incubation at 25 °C. The absorbance of the blank was subtracted from that of the sample and the cholinesterase inhibitory activity was expressed as galanthamine equivalents (mgGALAE/g extract).

For the Tyrosinase inhibitory activity assay: A sample solution (0.2-2 mg/ml) was mixed with tyrosinase solution (40 μL, Sigma) and phosphate buffer (100 μL, pH 6.8) in a 96-well microplate and incubated for 15 min at 25 °C. The reaction was then initiated with the addition of L-DOPA (40 μL, Sigma). Similarly, a blank was prepared by adding a sample solution to all reaction reagents without the enzyme (tyrosinase) solution. The sample and blank absorbances were read at 492 nm after a 10 min incubation at 25 °C. The absorbance of the blank was subtracted from that of the sample and the tyrosinase inhibitory activity was expressed as kojic acid equivalents (mgKAE/g extract).

For the α-amylase inhibitory activity assay: A sample solution (0.2-2 mg/ml) was mixed with an α-amylase solution (ex-porcine pancreas, EC 3.2.1.1, Sigma) (50 μL) in a phosphate buffer (pH 6.9 with 6 mM sodium chloride) in a 96-well microplate and incubated for 10 min at 37 °C. After pre-incubation, the reaction was initiated with the addition of a starch solution (50 μL, 0.05%). Similarly, a blank was prepared by adding a sample solution to all reaction reagents without the enzyme (α-amylase) solution. The reaction mixture was incubated for 10 min at 37 °C. The reaction was then stopped with the addition of HCl (25 μL, 1 M). This was followed by the addition of the iodine-potassium iodide solution (100 μL). The sample and blank absorbances were read at 630 nm. The absorbance of the blank was subtracted from that of the sample and the α-amylase inhibitory activity was expressed as acarbose equivalents (mmol ACE/g extract).

For the α-glucosidase inhibitory activity assay: A sample solution (0.2-2 mg/ml) was mixed with the glutathione (50 μL), α-glucosidase solution (from Saccharomyces cerevisiae, EC 3.2.1.20, Sigma) (50 μL) in a phosphate buffer (pH 6.8) and PNPG (4-N-trophenyl-α-D-glucopyranoside, Sigma) (50 μL) in a 96-well microplate and incubated for 15 min at 37 °C. Similarly, a blank was prepared by adding a sample solution to all reaction reagents without the enzyme (α-glucosidase) solution. The reaction was then stopped with the addition of sodium carbonate (50 μL, 0.2 M). The sample and blank absorbances were read at 400 nm. The absorbance of the blank was subtracted from that of the sample and the α-glucosidase inhibitory activity was expressed as acarbose equivalents (mmol ACE/g extract).

Table S1. HPLC–MS/MS acquisition parameters (dynamic MRM mode) used for the analysis of the 38 marker compounds.

No.	Compounds	Precursor ion, *m/z*	Product ion, *m/z*	Fragm-entor, V	Collision energy, V	Polarity	Retention time (Rt, min)
1	Gallic acid	169	125.2*	97	12	Negative	6.96
2	Neochlorogenic acid	353	191.2*, 179	82	12, 12	Negative	9.52
3	Delphinidin-3-galactoside	465.01	303*	121	20	Positive	11.36
4	(+)-Catechin	289	245.2*,109.2	131	8, 20	Negative	11.44
5	Procyanidin B2	576.99	576.99*, 321.2	160	0, 32	Negative	12.41
6	Chlorogenic acid	353	191.2*, 127.5	82	12, 20	Negative	12.42
7	*p*-Hydroxybenzoic acid	137	93.2*	92	16	Negative	12.86
8	(-)-Epicatechin	289	245.1*, 109.1	126	8, 20	Negative	13.03
9	Cyanidin-3-glucoside	449	287.3*, 255.6	121	20, 20	Positive	13.14
10	Petunidin-3-glucoside	479.01	317*, 302	121	20, 44	Positive	13.26
11	3-Hydroxybenzoic acid	137	93.2*	88	8	Negative	13.59
12	Caffeic acid	179	135.2*, 134.1	92	12, 24	Negative	13.65
13	Vanillic acid	167	152.4*, 108.1	88	12, 20	Negative	14.32
14	Resveratrol	227	185*, 143.2	136	12, 20	Negative	14.40
15	Pelargonidin-3-glucoside	433.01	271*, 121	116	24, 50	Positive	14.52
16	Pelagonidin-3-rutinoside	579.01	271*	145	32	Positive	14.56
17	Malvidin-3-galactoside	493.01	331*, 315.1	121	20, 50	Positive	14.64
18	Syringic acid	196.9	182.2*, 121.2	93	8, 12	Negative	15.28
19	Procyanidin A2	575	575*, 285	170	0, 20	Negative	16.18
20	*p*-Coumaric acid	163	119.2*, 93.2	83	12, 36	Negative	16.70
21	Ferulic acid	193	134.2*, 131.6	83	12, 8	Negative	17.10
22	3,5-Dicaffeoylquinic acid	514.9	353.1*, 191	117	8, 28	Negative	17.61
23	Rutin	609	300.2*, 271.2	170	32, 50	Negative	17.73
24	Hyperoside	465.01	303*, 61.1	97	8, 50	Positive	18.33
25	Isoquercitrin	463	271.2*, 300.2	155	44, 24	Negative	18.36
26	Delphinidin-3,5-diglucoside	462.9	300.1*	165	24	Negative	18.38
27	Phloridzin	435.39	273*, 167	155	8, 28	Negative	18.83
28	Quercitrin	446.99	300.2*, 301.2	160	24, 16	Negative	19.61
29	Myricetin	316.99	179.1*, 182	150	16, 24	Negative	19.61
30	Naringin	578.99	271.3*, 151.3	170	32, 44	Negative	19.62
31	Kaempferol-3-glucoside	447	284.2*, 255.2	170	24, 40	Negative	19.77
32	Hesperidin	611.01	303*, 334.8	112	20, 12	Positive	20.19
33	Ellagic acid	301	301*, 229	170	0, 24	Negative	21.41
34	*Trans*-Cinnamic acid	149	131.2*, 77.2	74	4, 36	Positive	21.44
35	Quercetin	300.99	151.2*, 179.2	145	16, 12	Negative	21.87
36	Phloretin	272.99	167*, 123	116	8, 20	Negative	22.30
37	Kaempferol	287.01	153*, 69.1	60	36, 50	Positive	23.84
38	Isorhamnetin	314.99	300.2*, 196.1	145	16, 4	Negative	24.57

* These product ions were used for quantification.

References

Grochowski, D. M., Uysal, S., Aktumsek, A., Granica, S., Zengin, G., Ceylan, R., . . . Tomczyk, M. (2017). In vitro enzyme inhibitory properties, antioxidant activities, and phytochemical profile of Potentilla thuringiaca. *Phytochemistry Letters, 20*, 365-372.

Uysal, S., Zengin, G., Locatelli, M., Bahadori, M. B., Mocan, A., Bellagamba, G., . . . Aktumsek, A. (2017). Cytotoxic and enzyme inhibitory potential of two Potentilla species (P. speciosa L. and P. reptans Willd.) and their chemical composition. *Frontiers in pharmacology, 8*, 290.
